# A UK single‐center pilot experience using a novel robotic inchworm colonoscopy system

**DOI:** 10.1002/deo2.70123

**Published:** 2025-04-29

**Authors:** Jabed F. Ahmed, Sergio Coda, Purushothaman Premchand, Saswata Banerjee, Nisha Patel

**Affiliations:** ^1^ The Hamlyn Center Imperial College London London UK; ^2^ Gastroenterology Imperial College Healthcare NHS Trust London UK; ^3^ Gastroenterology Barking, Havering & Redbridge University NHS Trust Ilford UK

**Keywords:** colonoscopy, discomfort, outpatients, pain, robotics

## Abstract

**Introduction:**

Colonoscopy is the gold standard investigation in the lower gastrointestinal tract. However, 75% of patients can experience pain with moderate sedation. The application of robotic technology aims to overcome difficulties faced including better utilization of rooms for advanced procedures and to achieve a complete colonoscopy in patients restricted by pain and technical challenges.

**Methods:**

This pilot study, the first at a UK‐National Health Service Hospital between January 2023 to August 2024 with one expert endoscopist performing the robotic colonoscopy (RC). Patients with failed previous standard colonoscopy (SC) along with index diagnostic procedures deemed potentially difficult were recruited. Procedures were performed outside the endoscopy unit similar to an outpatient clinical room.

**Results:**

Ninety‐three patients were recruited (41 men:52 women), mean age of 53.8 years over 20 months. The commonest indications for RC were rectal bleeding (26.9%), failed SC (22.6%), and change in bowel habits (17.2%). Twenty‐one patients had failed the previous SC with 14 patients achieving completion with subsequent RC (66% improvement). The average cecal intubation time of 41.07 min with an average total procedure time of 76.48 min. A significant improvement in patient discomfort score was reported (4.71 SC vs. 1.71 RC; *p* < 0.001).

**Conclusions:**

RC provides a significantly more comfortable colonoscopy and has great potential to improve safety in colonoscopy from this early cohort of patients. Direct visualization, biopsy, and polypectomy are still possible with RC. This study has demonstrated a viable alternative to SC. With no sedation it allows procedures to be conducted outside the traditional endoscopy unit such as outpatients. The study highlights a learning curve to reduce cecal intubation time.

## INTRODUCTION

Colonoscopy is the gold standard investigation for diagnosis and treatment in the lower gastrointestinal tract.^1^ Pre‐procedure screening with patients has placed the highest importance on comfort experienced.^2^ We also know increased completion rate in colonoscopy is vital in ensuring all polyps, adenomas, and colorectal cancer pick‐up is maximized.^3^ The current completion rate is 95.1% for the Bowel Cancer Screening Programme patients from the latest available government figures.^4^

Current issues in standard colonoscopy (SC) include incomplete procedures due to pain and discomfort or withdrawal of consent by the patient. This applies to mainly unsedated or moderately conscious sedation procedures. Failure of the operator to reach the caecum may be a result of unresolved looping restricting further advancement.^5^

A population‐based study reported a 13.1% incomplete rate for a cohort of 300,000 patients over a 5‐year period.^5^ The knock‐on effect can result in reduced engagement in surveillance and follow‐up with patients who require further care.^6^

There has been a move to performing endoscopic procedures outside the traditional endoscopy unit such as trans‐nasal‐endoscopy in the outpatient setting. This is also an option for disposable procedures which allows the potential of expanding the reach of delivering endoscopic services to rural or less affluent areas which may not be able to support a traditional setup with cleaning and reprocessing.^7^

The introduction of robotic technology has advanced and helped overcome some of the issues faced by the current standard of care. Disposable options are now commercially available such as Ambu.^8^ The design of the robotic system allows less pain and discomfort to be reported from a smoother, less forceful, and sometimes autonomous motion.^9^ Robotic systems have been shown to be a successful alternative to a previously failed procedure with a standard scope such as Aer‐O‐Scope with a study reporting a 98.2% cecal intubation rate (CIR) with no mucosal damage or adverse effects.^10^

**FIGURE 1:**
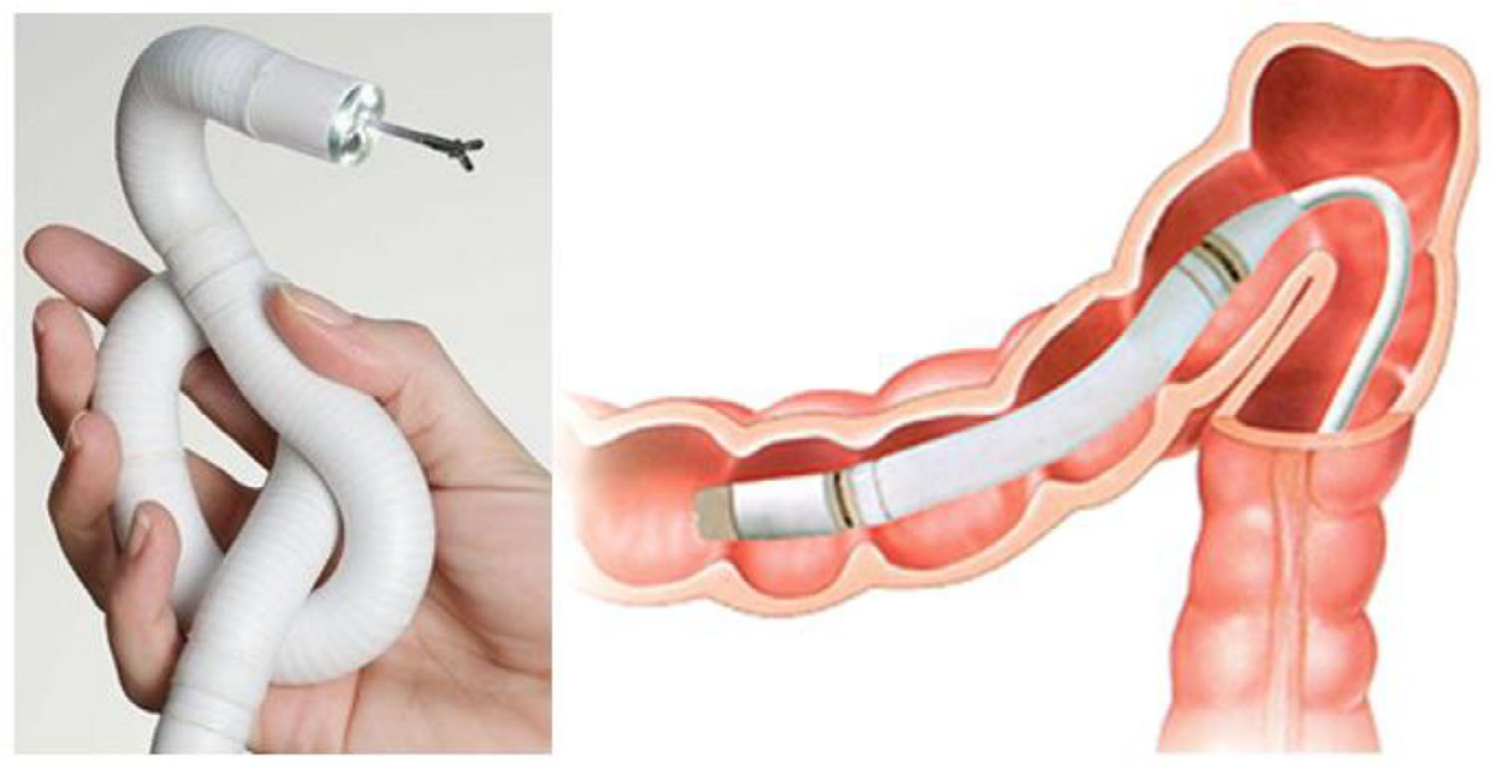
Endotics robotic colonoscope^13^ (Adapted from Ciuti et al., 2020).

**FIGURE 2:**
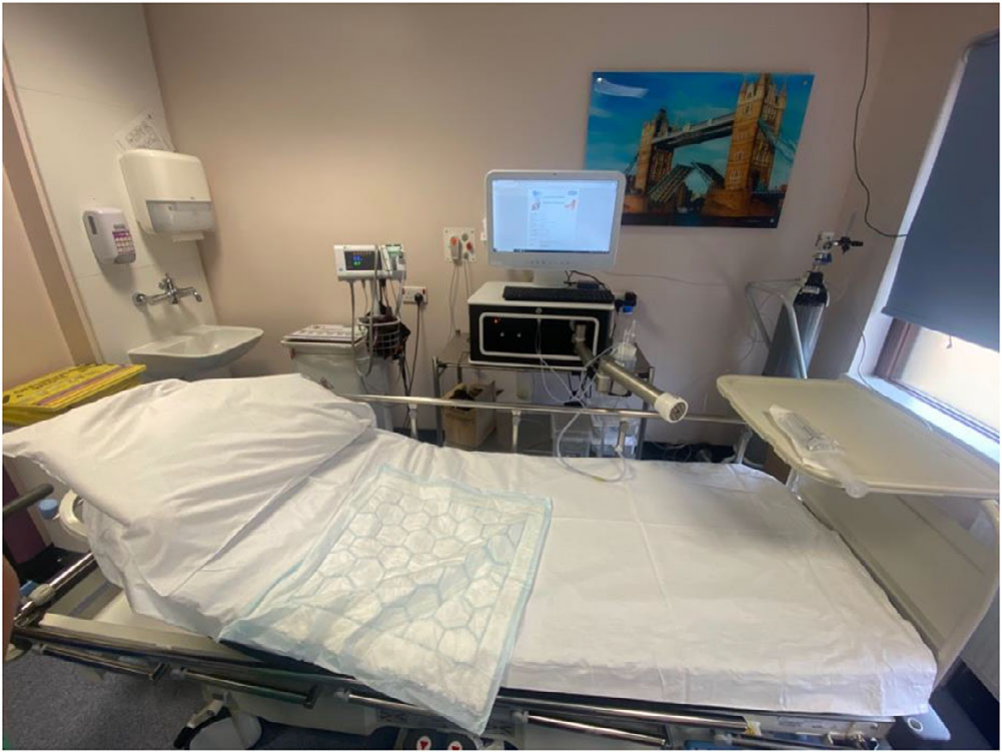
Procedure setup with computer monitor workstation, console, and patient bed.

**FIGURE 3:**
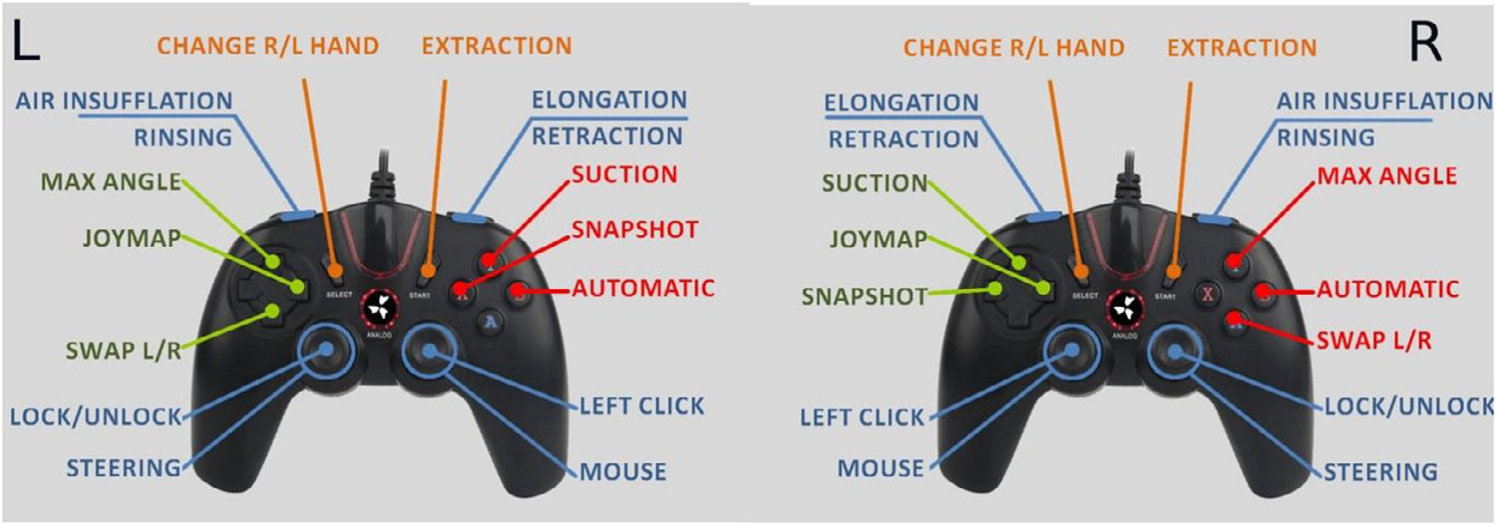
Hand‐held controller with a description of each button action. (Controller can be configured to dominant hand)

Endotics (Figure 1) is a CE‐marked (2017) and FDA‐approved (2020) robotic system for endoscopic use. It uses bio‐inspired principles such as contract‐and‐expand crawling, suction, and unsuction along a path to access difficult cavities and navigate internal organs, overcoming anatomical and technical challenges.^11^ The system consists of a disposable robotic colonoscope, a console that the robot attaches to, a workstation, and a remote controller (Figures 2 and 3).

Similarities with the SC include providing the same instructions to patients regarding bowel preparation. During the procedure utilizing the same position changes and biopsies are still possible. Sedation is also possible for patients, with the aim for reduced dosage given the anticipated lower pain and discomfort with Endotics.

The system can be set up in any hospital clinic room and is composed of a single‐use disposable soft robotic external device.

The first published study using Endotics was performed by Cosentino.^12^ This study highlighted the advantage of the system in patients who have failed SC.

Tumino undertook a head‐to‐head study, Endotics versus SC which reported noninferiority.^13^, ^14^ Trecca performed a pilot study showing the learning curve involved with the Endotics system. It reported individual learning progress.^15^ Consentino reported a cecalCIR of 92.3% in cases with a failed first procedure with SC. The study highlighted the importance of nursing staff and how it can impact performance for the endoscopist including reducing the learning curve for Endotics.^16^ Further studies have continued to improve the Endotics system with a 2023 study reporting on improved field‐of‐vision with a viewing angle of 110°.^17^ Moving robotic colonoscopy (RC) to ileocolonoscopy has also been demonstrated with Endotics, whilst difficult but still achievable as demonstrated by a published case in 2020.^18^

Barking, Havering and Redbridge National Health Service (NHS) Trust is the first UK center to undertake robotic endoscopy with NHS patients using the Endotics system.^19^ We report on the first cohort of patients undertaking this service in a single‐center pilot study.^20^

## METHODS

This prospective study has been conducted from January 2023 to August 2024 over a 20‐month period. One UK‐accredited expert (>1000 colonoscopies) endoscopist was trained in the Endotics system and has undertaken the RC procedures at one NHS Hospital Trust. Integrated Research Application System approval was obtained to perform this pilot study with local governance approval (Appendix S1).

The measurable objectives of the study include cecal intubation and total procedure time, completed procedures, and patient discomfort score.

Inclusion criteria include patients: (1) adult patients; (2) able to give informed consent, (3) undertaking a diagnostic procedure; and (4) undertaking their first or repeat colonoscopy procedure.

Exclusion criteria include patients: (1) with no consent; (2) recruited to another research study; and (3) undergoing a planned therapeutic colonoscopy or lower gastrointestinal bleed bleed.

Patients will be identified in outpatients who are listed for diagnostic colonoscopy and have risk factors possibly making them a difficult procedure such as previous abdominal surgery or known diverticular disease. Patients who have had a failed colonoscopy with SC due to pain or technical difficulty will also be recruited. Patients were contacted to inform them they are candidates for an RC and will potentially benefit over a SC with information on the advantages of the robotic technique.

On the day of the procedure, patients undergo a routine pre‐checklist with no additional pre‐procedure requirements for RC. Patients are consented and assessed for any nitrous oxide or sedation (fentanyl and midazolam).

They then undertake the RC procedure in an outpatient clinic room. The robotic colonoscope is placed into the anus with the patient in the left lateral position as per normal protocol.

**FIGURE 4:**
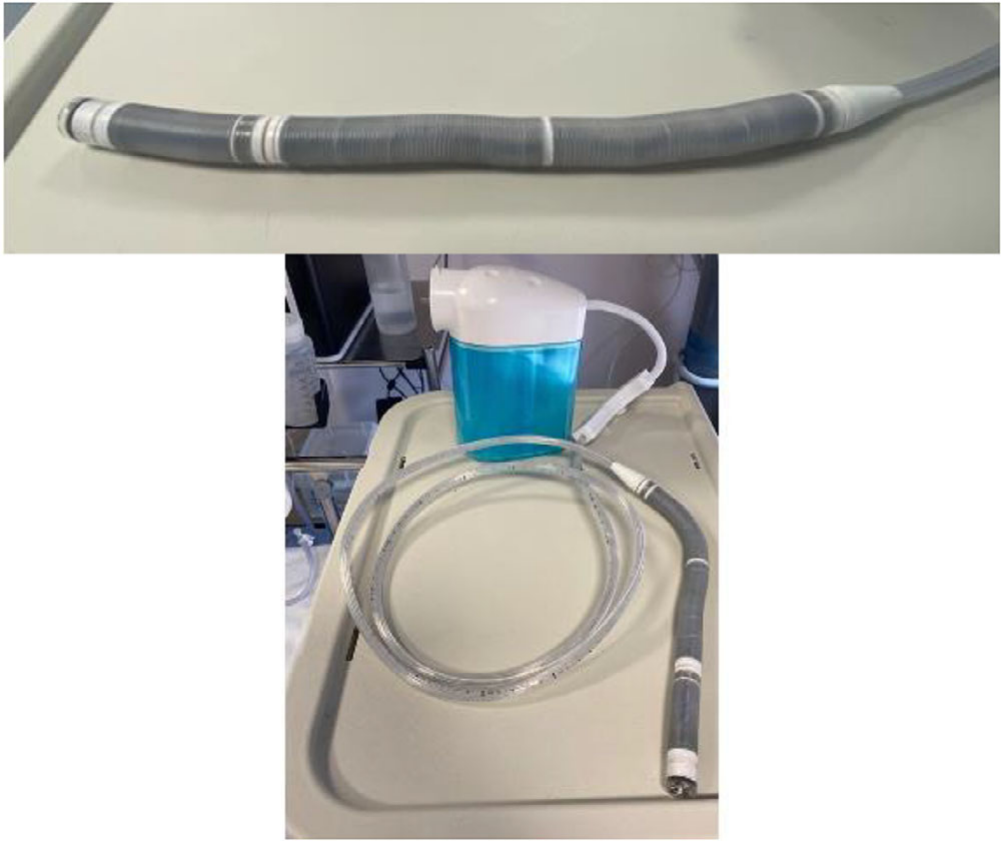
Disposable robotic colonoscope with disposable console attachments.

**FIGURE 5:**
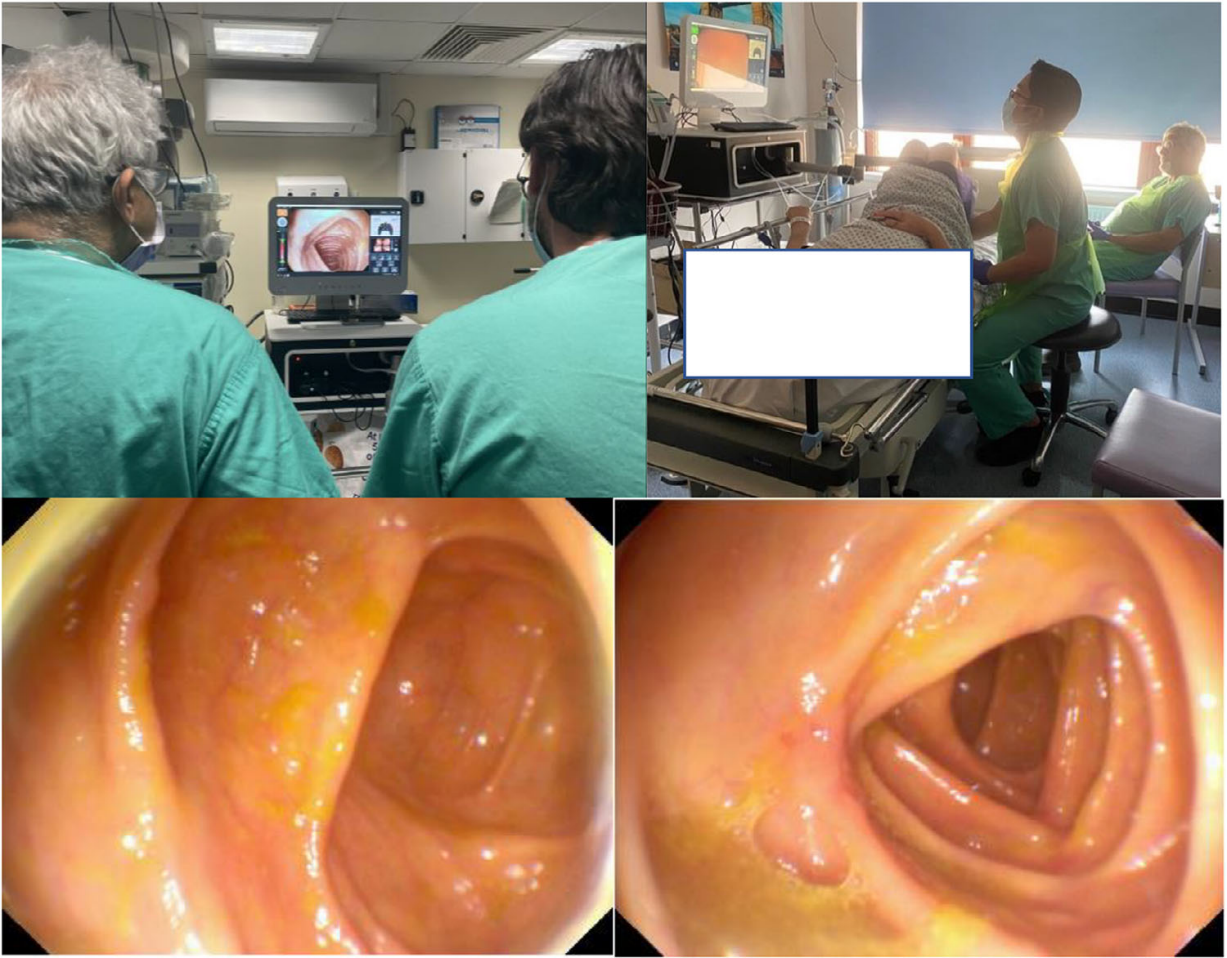
Endoscopist performing a procedure with patient and nurse assistant. Endotics camera images (resolution information not available).

The disposable robotic device has an active segment with a steerable tip, a flexible body, and a passive tail (Figure 4). The head houses a video system, consisting of a camera with a light source, a water irrigation channel, and one for air insufflation and suction. The endoscopist steers the robot colonoscope using the handheld controller with both hands whilst viewing in real‐time the robotic movement in the large bowel on the monitor (Figure 5).

The endoscopist is able to steer the robot with a 180° range of movement of the head in any direction. The body is able to be elongated allowing forward movement following the shape of the colon. The robot has flexibility and can adapt its shape to colonic bends (Appendix S2).

Any biopsies required are taken as per standard practice alongside any follow‐up that is required booked afterward as per SC.

Following the procedure, feedback is obtained from the patient on their experience including discomfort scores.

## RESULTS

### Demographics and indications

A total of 93 patients (41 men:52 women) with a mean age of 55 (20–81 range) were recruited for RC between January 2023 and August 2024.

Ten patients reported an abdominal surgical history. The most common indications for RC were PR‐bleeding (25%–26.9%), failure with SC (21%–22.6%), and change in bowel habits (16%–17.2%). Table 1 provides a summary of all indications from the patient cohort.

**TABLE 1:** Pilot study patient cohort indications.

Indication	Number of patients (% of total)
PR bleeding	25 (28.4%)
Failed standard colonoscopy	21 (23.9%)
Change in bowel habit	16 (18.2%)
CT virtual colonoscopy	7 (8%)
IBD	6 (6.8%)
Positive FCP	4 (4.5%)
Positive FIT	3 (3.4%)
Post CRC surveillance	2 (2.3%)
Anaemia	1 (1.1%)
Polyp follow up	1 (1.1%)
Acromegaly surveillance	1 (1.1%)
Diverticular disease	1 (1.1%)

### Previous SC

Twenty‐one of the 93 patients had undergone a previous SC which failed (Table 2). Seven of the 21 patients who had failed standard procedure, also failed to achieve completion with RC. Fourteen of the 21 achieved completion with RC which failed the previous SC producing a 66% improvement and completion for patients who required colonoscopy.

**TABLE 2:** Reasons for failure in previous standard colonoscopy procedures (*n* = 21).

Reason for failure	Number of standard colonoscopy procedures (% of total)
Patient pain (In all cases there was a withdrawal of consent in the sigmoid 5, descending 4, and transverse 5.	14 (66.7%)
Technical difficulty (Endoscopist unable to progress passed rectosigmoid or descending due to looping 5, severe diverticular disease not allowing progression 1).	6 (28.6%)
Poor views due to bowel preparation	1 (4.8%)

### Patient‐reported outcomes

The difference in pain score assessment for patients who underwent RC after failed SC is highlighted in Table 3. The mean discomfort score value for SC was 4.71 (SD: 0.73) and for RC was 1.71 (SD: 1.44). The paired T‐test for both interventions was 7.65. This suggests that RC significantly reduces discomfort compared to SC (*p* < 0.001).

**TABLE 3:** Patient discomfort score comparison (*n* = 14)

Patient (all previously failed standard colon procedure due to pain)	Standard colonoscopy discomfort score (0‐5)	Robotic Colonoscopy Discomfort score (0–5)
1	5	1
2	5	1
3	5	4
4	5	1
5	5	1
6	5	4
7	5	1
8	3	1
9	5	5
10	3	1
11	5	1
12	5	1
13	5	1
14	5	1

### Failed procedures

Nineteen of the 93 RC procedures were deemed incomplete (Table 4).

**TABLE 4:** Reasons for failure in robotic colonoscopy procedures (*n* = 19).

Reason for failure	Number of standard colonoscopy procedures (% of total)
Patient pain (In all cases there was a withdrawal of consent in the sigmoid/descending segment. Patients were not able to continue with the addition of nitrous oxide also)	10 (52.6%)
Technical difficulty (endoscopist unable to progress passed rectosigmoid due to tightness of segment, deemed not pathological 3, severe diverticular disease not allowing progression with the robot 4).	7 (36.8%)
Poor views due to bowel preparation	2 (10.5%)

### Outcomes

The endoscopist and nurse reported comfort scores (Table 5).

**TABLE 5:** Endoscopist and nurse reported comfort scores.

Endoscopist comfort description	Number of patients (% of total‐93 patients score recorded)	Nurse reported comfort score	Number of patients (% of total‐53 patients score recorded)
No pain/comfortable	31 (33.3%)	1	18 (34%)
Mild discomfort	47 (50.5%)	2	14 (26.4%)
Moderate discomfort	9 (9.7%)	3	12 (22.6%)
Severe discomfort	6 (6.5%)	4	8 (15.1%)
		5	1 (1.9%)

Premedication used is shown in Table 6.

**TABLE 6:** Premedication used in robotic colonoscopy (*n* = 103).

Premedication	Number of patients (% of total)
Nitrous oxide	21 (20.4%)
Sedation (fentanyl & midazolam)	1 (1%)
No premedication	81 (78.6%)

Bowel preparation scoring is shown in Table 7.

**TABLE 7:** Bowel preparation scoring in robotic colonoscopy (*n* = 93).

Bowel preparation	Number of patients (% of total)
Poor	2 (2.2%)
Fair	4 (4.3%)
Good	72 (77.4%)
Very good	6 (6.5%)
Excellent	9 (9.7%)

Robotic colonoscopy findings are shown in Table 8.

**TABLE 8:** Findings from robotic colonoscopy (*n* = 93).

Findings	Number of patients (% of total)
Diverticulosis	12 (12.9%)
Colitis	3 (3.2%)
Polyps	2 (2.2%)
Normal	53 (57%)
Incomplete	21 (22.6%)
Hemorrhoids	2 (2.2%)

### Key performance indicators

The mean average cecal intubation time was 41.07 min (range 15–90 min). The mean average total procedure time was 76.48 min (range 20–109 min). No adverse events were reported for the patients undertaking RC from the pilot study cohort.

## DISCUSSION

This pilot study is the first in the UK to perform RC on NHS patients. It has demonstrated a real‐world clinical implementation for RC.

Major advantages of this new system include the ability to perform endoscopy outside of the traditional endoscopy unit, allowing the release of vital endoscopy space for more complicated procedures requiring the specific endoscopy unit facilities. Access is greatly increased also by being able to use any outpatient room in a hospital and reduction in workforce needed such as recovery nurses, however, any involvement with nitrous oxide and sedation would need to ensure adequate staff and monitoring protocol is followed.

The single‐use soft robot attachment allows no reprocessing or cleaning requirements and with cost materials being inexpensive provides a more economical option for units.

The study has reported an improvement in completion in patients who had undergone a failed first‐line procedure with SC. This will allow better surveillance and follow‐up to be implemented and contribute to reducing post‐colonoscopy colorectal cancer rates.^21^

No sedation or a lower average sedation dosage was used for most RC patients. This has the advantage of quicker recovery time, less sedation‐related complications, and more streamlined post‐procedure nursing care.^22^

Subjective pain scoring was also significantly lower with less pain reported for the robotic procedure. Given the main patient perspective reason for non‐attendance and lack of engagement in services is due to perceived or anticipated pain, this will contribute to a greater follow‐up response rate and surveillance attendance which will again contribute to the reduction of post‐colonoscopy colorectal cancer rates.^23^

Although no formal study has yet to validate, the learning curve anecdotally appears to be shorter with RC due to the joystick controller setup. This is in part explained by the improved controller interface of technology and in part by users gaining collateral controller experience such as those who have played computer games and have greater experience with hand‐eye visual technology. The improved controller interface of medical devices also allows hand discrimination to be removed with the ability to change the configuration of buttons on the initial setup.

The ergonomic advantages of the controller are also beneficial to the health and wellbeing of the endoscopist with reduced neck and shoulder issues attributed to years of colonoscopy procedures.^24^ Grip force required is also reduced withholding of a controller compared to the standard colonoscope. The existing issues of restricted or reduced access to adjust dial controls due to the range of finger movement of the user are removed for users with small hand sizes. Fatigue and subsequent reduced quality of endoscopic assessment is also prevented which has been demonstrated with users in SC with the use of a controller.^25^

The added financial cost of purchasing, cleaning, and reprocessing standard colonoscopes can be as much as $500 per scope per use with further costs for repairs and maintenance.^26^, ^27^ Disposable colonoscopes allow a cost saving for endoscopy units and can be relatively inexpensive when comparing material costs.^28^ The approximate cost per unit for this pilot study is £330 per patient.

Despite this not being a head‐to‐head study such as Tumino et al, this study represents a more updated evaluation of the robotic system given the advancement in the SC technology and in clinicians' technical abilities when using a standard scope. The head‐to‐head study was also taken in patients with a higher threshold for inclusion as patients with a familial or clinical risk of polyps were evaluated. This may partially explain a higher cecalCIR in this study compared to this pilot study.

The main disadvantage identified from this study is the significantly higher cecal intubation and total procedure time. This can result in a lower number of patients having the procedure on a given set endoscopy list weakening the argument for an efficient screening tool and also reducing service output. The completion rate is also below 80% for both index and follow‐on from SC procedures. CIR was 83.3% for the 72 patients undertaking an index colonoscopy which is relatively low when compared to expected key performance indicator (KPI) targets for colonoscopy and CIR. The factors contributing to this include a higher chance of a technically difficult procedure based on the patient recruitment screening. Patients selected had a failed SC or had risk factors identified such as diverticular disease.

The main adjustable factor to completion rate and procedure time will be the learning curve for the user performing RC with an expectation to improve both performance objectives with further procedures the endoscopist undertakes as they move along and improve on the learning curve. This was not yet observed in this pilot study consistently when observing subsequent procedures. The controller interface will also contribute to the improvement of performance indicators.

A pilot study by Trecca reported the cecal intubation time decreased from 55 to 22 min following a learning period involving 27 patients.^19^ It would be valuable to investigate the learning curve specifically for patients with previously incomplete SC to understand how experience impacts procedural efficiency in these challenging cases.

There is a small limitation on intervention options available with RC. Whilst biopsies, applying tattoo and polypectomy up to 1 cm is possible, polyps greater than 1 cm or multiple polyp removal is currently technically difficult and may require a reversion back to an SC and is a significant limitation to the robotic technique.

The current setup for the pilot study has only one expert endoscopist trained in the technique limiting its widespread utilization. The long‐term advancement will require further users to be trained and undertake the procedure regularly. Introducing the technique at a trainee level to endoscopists could also prove beneficial. This will allow the expansion of the service and increase the number of patients benefiting. New users may also be able to enter the learning curve at a more advanced stage thanks to the initial users and achieve competency and performance indicator thresholds sooner.

In addition to researching the role of RC in rescuing difficult cases, assessment of detection rates for various polyp morphologies will be of value for quality assessment. In this pilot study polyp detection rate (PDR) was 2% (2/93) which is significantly lower than the expected KPI for a diagnostic colonoscopy cohort. This will be a result of a small pilot cohort and skewed selection with patients being selected of whom had polypectomy in their first standard procedure. The low CIR as discussed; due to the endoscopist learning curve will also contribute to potentially missed polyps and a low PDR.

Further information on patients’ perception of pain with a self‐reported questionnaire and the use of a visual analog scale scoring for discomfort would provide valuable insight and improve comparable analysis on this new technology and is an area of future work.

A follow‐up to this pilot study will be a controlled trial of patients undergoing screening colonoscopy or screening/diagnostic colonoscopy and performing a statistical comparison between SC and RC. This was not possible at this stage due to the limited patient recruitment available and therefore direct comparison of key performance indicators and quality measures was not feasible. An additional future study will be comparing patients who fail an SC with receiving a second SC with an expert versus a robotics colonoscopy and comparing the difference in metrics.

## CONCLUSION

Robotic colonoscopy provides a significantly more comfortable colonoscopy and has great potential to improve safety in colonoscopy from this early cohort of patients. It provides an alternative to conventional colonoscopy with an opportunity to improve the completion rate such as those who had previously failed an SC providing optimism for non‐inferiority with this novel method. The ability to use the outpatient setting allows space in the endoscopy unit for other priority procedures. In addition to a minimal requirement for sedation and the removal of reprocessing with a fully disposable endoscope.

A learning curve is being established with an aim to reduce the total procedure time and to allow new users to reach clinical efficiency and competency sooner.

## CONFLICT OF INTEREST STATEMENT

None.

## ETHICS STATEMENT

Approval of the research protocol by IRAS (Integrated Research Application System). ID: 330829

## PATIENT CONSENT STATEMENT

N/A

## CLINICAL TRIAL REGISTRATION

N/A.

## Supporting information



FIGURE S1: principles of the movement for the endotics system^24^ (Adapted from Seah et al., 2017).

FIGURE S2: study design.

## References

[1] Rees CJ , Thomas Gibson S , Rutter MD *et al*. UK key performance indicators and quality assurance standards for colonoscopy. Gut 2016; 65: 1923–9. 27531829 10.1136/gutjnl-2016-312044 PMC5136732

[2] Tierney M , Bevan R , Rees CJ *et al*. What do patients want from their endoscopy experience? The importance of measuring and understanding patient attitudes to their care. Frontline Gastroenterol 2016; 7: 191–8. 27429733 10.1136/flgastro-2015-100574 PMC4941156

[3] Ishtiaq R , Zulfiqar L , Gangwani MK , Aziz M . Adenoma detection rate vs. adenoma per colonoscopy as quality indicators for colon cancer screening. Transl Gastroenterol Hepatol 2023; 8: 24. 37601737 10.21037/tgh-22-92 PMC10432231

[4] UK Government . Bowel cancer screening annual report 2021 to 2022 *[Internet]* . 2024. Available from: https://www.gov.uk/government/publications/bowel‐cancer‐screening‐annual‐report‐2021‐to‐2022/bowel‐cancer‐screening‐annual‐report‐2021‐to‐2022

[5] Shah HA , Paszat LF , Saskin R , Stukel TA , Rabeneck L . Factors associated with incomplete colonoscopy: A population‐based study. Gastroenterology 2007; 132: 2297–303. 17570204 10.1053/j.gastro.2007.03.032

[6] Brahmania M , Park J , Svarta S , Tong J , Kwok R , Enns R . Incomplete colonoscopy: Maximizing completion rates of gastroenterologists. Can J Gastroenterol 2012; 26: 589–92. 22993727 10.1155/2012/353457 PMC3441163

[7] Lim S , Haboubi HN , Anderson SHC *et al*. Transnasal endoscopy: Moving from endoscopy to the clinical outpatient‐blue sky thinking in oesophageal testing. Frontline Gastroenterol 2022; 13: e65–71. 35812036 10.1136/flgastro-2022-102129 PMC9234731

[8] Ambu . Single use medical products *[Internet]* . 2024. Available from: https://www.ambu.co.uk/products?gad_source=1&gclid=Cj0KCQjwzva1BhD3ARIsADQuPnXYXSa_HZF6i93cX5SnLcdSVQnwL2mouvrwVabxi‐OfG6qYcZZnV_0aAo41EALw_wcB

[9] Yeung CK , Cheung JL , Sreedhar B . Emerging next‐generation robotic colonoscopy systems towards painless colonoscopy. J Dig Dis 2019; 20: 196–205. 30834714 10.1111/1751-2980.12718 PMC6849516

[10] Ciuti G , Skonieczna‐Żydecka K , Marlicz W *et al*. Frontiers of robotic colonoscopy: A comprehensive review of robotic colonoscopes and technologies. J Clin Med 2020; 9: 1648. 32486374 10.3390/jcm9061648 PMC7356873

[11] Ahmed JF , Franco E , Rodriguez Y . Baena F , Darzi A , Patel N . A review of bioinspired locomotion in lower GI endoscopy. Robotica 2024; 42: 1–11

[12] Cosentino F , Tumino E , Passoni GR , Morandi E , Capria A . Functional evaluation of the Endotics system, a new disposable self‐propelled robotic colonoscope: In vitro tests and clinical trial. Int J Artif Organs 2009; 32: 517–27. 19844894 10.1177/039139880903200806

[13] Tumino E , Sacco R , Bertini M , Bertoni M , Parisi G , Capria A . Endotics system vs colonoscopy for the detection of polyps. World J Gastroenterol 2010; 16: 5452–6. 21086563 10.3748/wjg.v16.i43.5452 PMC2988238

[14] Tumino E , Parisi G , Bertoni M *et al*. Use of robotic colonoscopy in patients with previous incomplete colonoscopy. Eur Rev Med Pharmacol Sci 2017; 21: 819–26. 28272700

[15] Trecca A , Catalano F , Bella A , Borghini R . Robotic colonoscopy: Efficacy, tolerability and safety. Preliminary clinical results from a pilot study. Surg Endosc 2020; 34: 1442–50. 31932942 10.1007/s00464-019-07332-6

[16] Cosentino F . Robotic colonoscopy endotics with colon wash: Two performing technologies in a winning combination. World J Gastroenterol Hepatol Endosc 2021; 3: 1–9

[17] Tumino E , Visaggi P , Bolognesi V *et al*. Robotic colonoscopy and beyond: Insights into modern lower gastrointestinal endoscopy. Diagnostics 2023; 13: 2452. 37510196 10.3390/diagnostics13142452 PMC10378494

[18] Trecca A , Catalano F , Borghini R . Robotic colonoscopy in a case of severe dolichocolon and the first case of robotic ileoscopy. Tech Coloproctol 2020; 24: 603–4. 32248364 10.1007/s10151-020-02205-w

[19] Vitramed . Robotic endoscopy *[Internet]* . 2022. Available from: https://vitramed.com/product/robotic‐endoscopy/#:~:text=The%20Endotics%20system%20consists%20of,without%20the%20need%20for%20pushing

[20] NHS Barking Havering and Redbridge University Hospital . Countries first robotic colonoscopy machine revealed *[Internet]* . 2023. Available from: https://www.bhrhospitals.nhs.uk/news/countrys‐first‐robotic‐colonoscopy‐machine‐unveiled‐3975/)

[21] Anderson R , Burr NE , Valori R . Causes of Post‐colonoscopy colorectal cancers based on World Endoscopy Organization System of Analysis. Gastroenterology 2020; 158: 1287–99. 31926170 10.1053/j.gastro.2019.12.031

[22] Welchman S , Cochrane S , Minto G *et al*. Systematic review: The use of nitrous oxide gas for lower gastrointestinal endoscopy. Aliment Pharmacol Ther 2010; 32: 324–33. 20491748 10.1111/j.1365-2036.2010.04359.x

[23] Hadaib I , Anglade P , Ibrahim H . Factors contributing to non‐attendance of gi endoscopic procedures in a tertiary care center in the Middle East. Asian Pac J Cancer Prev 2022; 23: 33–7. 35092369 10.31557/APJCP.2022.23.1.33 PMC9258662

[24] Al‐Rifaie A , Gariballa M , Ghodeif A , Hodge S , Thoufeeq M , Donnelly M . Colonoscopy‐related injury among colonoscopists: An international survey. Endosc Int Open 2021; 9: E102–9. 33403242 10.1055/a-1311-0561 PMC7775804

[25] Dong Z , Wang J , Chen Y *et al*. Negative effects of endoscopists' fatigue on colonoscopy quality on 34,022 screening colonoscopies. J Gastrointestin Liver Dis 2021; 30: 358–65. 34551036 10.15403/jgld-3687

[26] Larsen S , Kalloo A , Hutfless S . The hidden cost of colonoscopy including cost of reprocessing and infection rate: The implications for disposable colonoscopes. Gut 2020; 69: 197–200. 31413166 10.1136/gutjnl-2019-319108 PMC6984057

[27] Boston Scientific . A Glimpse at the True Cost of Reprocessing Endoscopes *[Internet]* . 2017. Available from: https://www.bostonscientific.com/content/dam/bostonscientific/uro‐wh/portfolio‐group/LithoVue/pdfs/Sterilization‐Resource‐Handout.pdf

[28] Ciocîrlan M . Low‐cost disposable endoscope: Pros and cons. Endosc Int Open 2019; 7: E1184–6. 31479502 10.1055/a-0959-6003 PMC6715437

