# Supplementary material for: A UK single‐center pilot experience using a novel robotic inchworm colonoscopy system
Source: DEN Open. 2025 Apr 29;6(1):e70123. doi: 10.1002/deo2.70123 (PMC12038173; doi:10.1002/deo2.70123)
Supplement: Supplementary file 1 — FIGURE S1: principles of the movement for the endotics system 24 (Adapted from Seah et al., 2017). [file DEO2-6-e70123-s002.docx]

**Appendix 1 - Detailed description of Endotics soft robot movement:**

A semi-automatic sequence of actions allows the robot to advance like an inchworm. This locomotion is achieved by two clampers located in the proximal and in the distal part of the probe. They grip the large bowel mucosa using a vacuum technique and a mechanical grasping action. The semi-automatic sequence can be described as follows: (Adapted from Consentino 2009)^16^ (Supporting Figure 1).

1. Clamper in the proximal part of the probe adheres to the mucosa (automatic phase)
2. The central part of the probe body is elongated by the endoscopist under visual control (manual phase)
3. Distal clamper adheres to the mucosa (automatic phase)
4. Proximal clamper is released (automatic phase)
5. Central part of the body is shortened (automatic phase)
6. Proximal clamper adheres to the mucosa (automatic phase)
7. Distal clamper is released (automatic phase); Such sequence is repeated several times in order to advance the probe through the colonic lumen


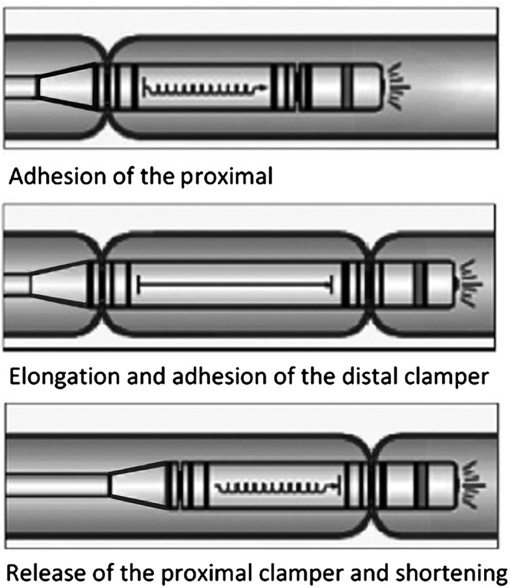


**Supporting Figure 1: Principles of the movement for the Endotics system^24^ (Adapted from Seah et al 2017)**
