# Supplementary figures and images for: A UK single‐center pilot experience using a novel robotic inchworm colonoscopy system
Source: DEN Open. 2025 Apr 29;6(1):e70123. doi: 10.1002/deo2.70123 (PMC12038173; doi:10.1002/deo2.70123)

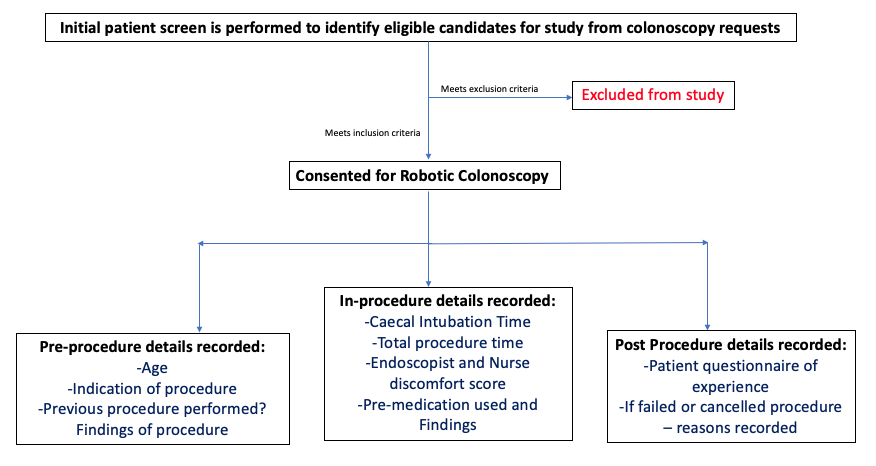


**Appendix 2: Study Design**

Supplement: Supplementary file 2 — FIGURE S2: study design. [file DEO2-6-e70123-s001.docx]
